# The relationship of leaf photosynthetic traits – *V*_cmax_ and *J*_max_ – to leaf nitrogen, leaf phosphorus, and specific leaf area: a meta-analysis and modeling study

**DOI:** 10.1002/ece3.1173

**Published:** 2014-07-25

**Authors:** Anthony P Walker, Andrew P Beckerman, Lianhong Gu, Jens Kattge, Lucas A Cernusak, Tomas F Domingues, Joanna C Scales, Georg Wohlfahrt, Stan D Wullschleger, F Ian Woodward

**Affiliations:** 1 Department of Animal and Plant Sciences, University of SheffieldAlfred Denny Building, Western Bank, Sheffield, S10 2TN, UK; 2 Environmental Sciences Division, Climate Change Science Institute, Oak Ridge National LaboratoryOak Ridge, Tennessee, 37831-6301; 3 Max Plank Institute for BiogeochemistryJena, Germany; 4 Department of Marine and Tropical Biology Cairns, James Cook UniversityCairns, Queensland, 4878, Australia; 5 Depto. de Biologia, Faculdade de Filosofia, Ciências e Letras de Ribeirão PretoAv. Bandeirantes, 3900 – CEP, 14040-901, Ribeirão Preto, Brasil; 6 Plant Biology and Crop Science, Rothamsted ResearchHarpenden, Herts, AL5 2JQ, UK; 7 University of Innsbruck, Institute of EcologySternwartestrasse 15, 6020, Innsbruck, Austria

**Keywords:** Carbon assimilation, carbon cycle, carboxylation, DGVM, electron transport, Farquhar model, land surface model, meta-analysis, mixed-effect multiple regression, noncarbon photosynthesis, TBM

## Abstract

Great uncertainty exists in the global exchange of carbon between the atmosphere and the terrestrial biosphere. An important source of this uncertainty lies in the dependency of photosynthesis on the maximum rate of carboxylation (*V*_cmax_) and the maximum rate of electron transport (*J*_max_). Understanding and making accurate prediction of C fluxes thus requires accurate characterization of these rates and their relationship with plant nutrient status over large geographic scales. Plant nutrient status is indicated by the traits: leaf nitrogen (N), leaf phosphorus (P), and specific leaf area (SLA). Correlations between *V*_cmax_ and *J*_max_ and leaf nitrogen (N) are typically derived from local to global scales, while correlations with leaf phosphorus (P) and specific leaf area (SLA) have typically been derived at a local scale. Thus, there is no global-scale relationship between *V*_cmax_ and *J*_max_ and P or SLA limiting the ability of global-scale carbon flux models do not account for P or SLA. We gathered published data from 24 studies to reveal global relationships of *V*_cmax_ and *J*_max_ with leaf N, P, and SLA. *V*_cmax_ was strongly related to leaf N, and increasing leaf P substantially increased the sensitivity of *V*_cmax_ to leaf N. *J*_max_ was strongly related to *V*_cmax_, and neither leaf N, P, or SLA had a substantial impact on the relationship. Although more data are needed to expand the applicability of the relationship, we show leaf P is a globally important determinant of photosynthetic rates. In a model of photosynthesis, we showed that at high leaf N (3 gm^−2^), increasing leaf P from 0.05 to 0.22 gm^−2^ nearly doubled assimilation rates. Finally, we show that plants may employ a conservative strategy of *J*_max_ to *V*_cmax_ coordination that restricts photoinhibition when carboxylation is limiting at the expense of maximizing photosynthetic rates when light is limiting.

## Introduction

Photosynthesis is the proximal driver of the carbon cycle (Canadell et al. 2007; Cadule et al. 2010) and is thus a core driver of carbon flux and central to carbon cycle models (e.g., Woodward et al. 1995; Cox 2001; Sitch et al. 2003; Zaehle and Friend 2010; Bonan et al. 2011). Enzyme kinetic models of leaf photosynthesis (Farquhar et al. 1980; described below) are typically embedded in global carbon cycle models to mechanistically reflect plant physiological responses to atmospheric CO_2_. The Farquhar et al. (1980) photosynthetic submodel and its subsequent variants (Von Caemmerer and Farquhar 1981; Farquhar and Wong 1984; Collatz et al. 1991; Harley et al. 1992) are at the heart of almost all land surface models of carbon flux, several ecosystem dynamic models, and dynamic global vegetation models. We hereafter refer to these global land surface, ecosystem, and vegetation models as terrestrial biosphere models (TBMs).

Simulated photosynthetic rates in TBMs are highly sensitive to *V*_cmax_ and *J*_max_ (Zaehle et al. 2005; Bonan et al. 2011; Verheijen et al. 2012), the maximum rate parameters of enzyme kinetic processes driving photosynthesis. Accuracy in these parameters is central to an effective photosynthetic submodel in the TBMs. Theory and empirical data suggest that these photosynthetic rates scale with leaf nitrogen (N) via the large amount of leaf N invested in the ribulose 1-5-bisphosphate oxygenase/carboxylase (RuBisCO) protein, and phosphorus (P) availability influences many aspects of plant physiology central to photosynthesis, including membrane solubility, ATP, and NADPH production (Marschner 1995; Taiz and Zeiger 2010). *V*_cmax_ and *J*_max_ have also been linked to structural leaf traits via specific leaf area (SLA). Theory and data (Kattge et al. 2009; Domingues et al. 2010; Cernusak et al. 2011) clearly suggest mechanistic links between *V*_cmax_, *J*_max_, and several functional plant traits that correlate with photosynthetic biochemistry.

Accurate simulation of plant physiological responses to atmospheric CO_2_ in TBMs thus requires data on how *V*_cmax_ and *J*_max_ scale with plant traits N, P, and SLA accounting for the immense species-specific and regional variation in availability of N and P and subsequent variation in leaf N, P, and SLA.

Here, we provide a global assessment of the relationship between *V*_cmax_ and *J*_max_ and leaf N, P, and SLA, drawing on estimates made on 356 species around the world.

### When do *V*_cmax_ and *J*_max_ variation matter?

TBMs typically assign a single, fixed *V*_cmax_ or *J*_max_ parameter value (Rogers 2014) to each plant functional type (PFT). Scaling from plant to ecosystem or globe is achieved via PFT distribution maps. Recently, however, the predictive performance of such models has improved by allowing parameter values to vary. For example, at sites of the FLUXNET network where high-resolution data exist on all parameters and rates, predictive performance improved when *V*_cmax_ and *J*_max_ were allowed to vary interannually (Groenendijk et al. 2011). Additionally, some TBMs improve prediction by simulating leaf nitrogen as part of the model and specify a linear relationship between *V*_cmax_ and leaf N (e.g., Woodward et al. 1995), defined for each PFT (Kattge et al. 2009). Finally, Mercado et al. (2011) demonstrated considerable improvements to model predictions of carbon fluxes in the Amazon when leaf P was taken into account.

Empirically, there is also a strong relationship between *J*_max_ and *V*_cmax_ (Wullschleger 1993; Beerling and Quick 1995), and most TBMs simulate *J*_max_ as a linear function of *V*_cmax_. However, this assumption could be erroneous because the correlation between *J*_max_ and *V*_cmax_ is likely to be influenced by leaf N, P, and SLA. The coordination hypothesis of photosynthetic resource allocation (Chen et al. 1993) states that the Calvin–Benson cycle limited rate of assimilation (*W*_*c*_, see below) equals the electron transport-limited rate of assimilation (*W*_*j*_). The relationship between *J*_max_ and *V*_cmax_ affects the relationship between *W*_*c*_ and *W*_*j*_ and may reflect coordination of these two rate-limiting biochemical cycles. When carboxylation is limiting photosynthesis, high investment in *J*_max_ relative to *V*_cmax_ would lead to electron transport not used in photosynthesis requiring dissipation of that energy to avoid photoinhibition (Powles 1984; Krause et al. 2012). However, when light is limiting photosynthesis, high investment in *J*_max_ relative to *V*_cmax_ would maximize photosynthetic rates. Therefore, a trade-off exists in high investment in *J*_max_ relative to *V*_cmax_ whereby the marginal benefit to photosynthetic rates when light is limiting is offset by the cost of energy dissipation when carboxylation is limiting.

### Moving forward: global variation in *V*_cmax_ and *J*_max_ as a function of N, P, and SLA

As noted above, we make here a global assessment of the relationship between *V*_cmax_ and *J*_max_ and leaf N, P, and SLA, drawing on estimates made on 356 species by treatment combinations around the world from 24 different studies. We used these data to test several hypotheses. First, we hypothesized that leaf P will modify the relationship of *V*_cmax_ to leaf N. Second, we hypothesized that leaf P will modify the relationship of *J*_max_ to *V*_cmax_. Third, drawing on the coordination hypothesis of photosynthetic resource allocation, we predict that the relationship between *J*_max_ and *V*_cmax_ results from efficient resource investment in *J*_max_ reflecting the trade-off between photosynthetic gain and costs of energy dissipation.

To test our hypotheses, we combine a global meta-analysis of the relationships of *V*_cmax_ and *J*_max_ with N, P, and SLA and then examine the consequences of these patterns in a leaf photosynthesis model. Combined, our effort offers a global-scale definition of *V*_cmax_ and *J*_max_ variation in relation to leaf-trait variation and provides an empirical alternative to single value PFT scaling or the type of tuned relationships presented above in global TBMs. Our empirical representation of *V*_cmax_ and *J*_max_ should lead to improved simulation of carbon fluxes across multiple scales.

## Materials and Methods

### Literature review & data collection

In September 2012, we searched the Thompson Reuters Web of Science database for “photosynthesis” or “carboxylation” and either “N,” “P,” or “SLA” and similar related search terms. The aim was to find papers that had simultaneously measured as many of the following leaf traits: *V*_cmax_, *J*_max_, leaf N, leaf P, and specific leaf area (SLA) or leaf mass-to-area ratio (LMA). Data were copied from tables or digitized from graphics using Grab It! (Datatrend Software 2008). Minimum requirements for inclusion in this study were that either *V*_cmax_ or *J*_max_ were calculated from A/C_*i*_ curves along with two of the other three leaf traits, yielding data from 24 papers and 135 species x location combinations, distributed globally (Tables 1 and S1). Some of these data were collected on plants in their natural environment and subject to natural environmental variation, while other data were collected on laboratory-grown plants (mostly tree species) subjected to experimental treatments. The majority of the species used in the greenhouses and laboratories were native to the area of the research center. Either species means or treatment means were collected leading to a dataset of 356 species/treatment combinations. The data can be downloaded from the ORNL DAAC (http://dx.doi.org/10.3334/ORNLDAAC/1224).

**Table 1:** Sources of data collected for the meta-analysis and associated information including location, number of species and any experimental treatment

Reference	Number of species	PFT*	Longitude (°E)	Latitude (°N)	Elevation (m)	Location	Country	Experiment	N	P
Aranda et al. (2005)	1	Temp Ev Bl	−3.43	39.23	650	Alburquerque	Spain	Light*water	Y	N
Bauer et al. (2001)	6	Temp Dc Bl and Ev Nl	−71.03	42.21	40	Havard forest	USA	CO_2_*N	Y	N
Bown et al. (2007)	1	Temp Ev Nl	176.13	−38.26	600	Purokohukohu Experimental Basin	NZ	N*P	Y	Y
Brück and Guo (2006)	1	Temp legume crop	10.08	54.19	40	Kiel	Germany	NH_4_ vs. NO_3_	Y	N
Calfapietra (2005)	1	Temp Dc Bl	11.48	42.22	150	Viterbo	Italy	CO_2_*N canopy level	Y	N
Carswell et al. (2005)	4	Temp Dc Bl and Ev Nl	170.3	−43.2	90	Okarito	NZ	N*P	Y	Y
Cernusak et al. (2011)	2	Trop Ev Bl	139.56	−22.59	150	Boulia	Australia	None	Y	Y
Cernusak et al. (2011)	2	“	133.19	−17.07	230	Sturt plains	Australia	None	Y	Y
Cernusak et al. (2011)	2	“	132.22	−15.15	170	Dry creek	Australia	None	Y	Y
Cernusak et al. (2011)	2	“	131.23	−14.09	70	Daly river	Australia	None	Y	Y
Cernusak et al. (2011)	2	“	131.07	−13.04	80	Adelaide river	Australia	None	Y	Y
Cernusak et al. (2011)	2	“	131.08	−12.29	40	Howard springs	Australia	None	Y	Y
Deng (2004)	2	Sub-trop forb	113.17	23.08	10	Guanzhou	China	None	Y	N
Domingues et al. (2010)	3	Trop Dc Bl	−1.5	15.34	280–300	Hombori	Mali	None	Y	Y
Domingues et al. (2010)	7	“	−1.17	12.73	250	Bissiga	Burkina Faso	None	Y	Y
Domingues et al. (2010)	8	“	−3.15	10.94	300	Dano	Burkina Faso	None	Y	Y
Domingues et al. (2010)	5	“	−1.86	9.3	370	Mole	Ghana	None	Y	Y
Domingues et al. (2010)	8	“	−1.18	7.3	170	Kogye	Ghana	None	Y	Y
Domingues et al. (2010)	21	Trop Dc Bl and Ev Bl	−1.7	7.72	200	Boabeng Fiame	Ghana	None	Y	Y
Domingues et al. (2010)	4	“	−2.45	7.14	25	Asukese	Ghana	None	Y	Y
Grassi (2002)	1	Sub-trop Ev Bl	149.07	−35.18	600	Canberra	Australia	N	Y	N
Han et al. (2008)	1	Temp Ev Nl	138.8	35.45	1030	Canberra	Australia	N	Y	N
Katahata et al. (2007)	1	Ev shrub	138.4	36.51	900	Niigata	Japan	Light*leaf age	Y	N
Kubiske (2002)	2	Temp Bl Dc	−84.04	45.33	215	Pellston	USA	N* CO_2_*light	Y	N
Manter (2005)	1	Temp Ev Nl	−122.4	45.31	75	Portland	USA	N	Y	N
Merilo et al. (2006)	2	Temp Ev Nl	26.55	58.42	65	Saare	Estonia	Light	Y	N
Midgley et al. (1999)	4	Temp Ev shrub	20	−34.5	120	Cape Agulhas	SA	CO_2_*N&P	Y	N
Porte and Lousteau (1998)	1	Temp Ev Nl	−0.46	44.42	60	Bordeaux	France	Leaf age*canopy level	Y	Y
Rodriguez-Calcerrada et al. (2008)	2	Temp Dc Bl	−3.3	41.07	50	Madrid	Spain	Light	Y	N
Sholtis (2004)	1	Temp Dc Bl	−84.2	35.54	230	Oak Ridge	USA	CO_2_*canopy level	Y	N
Tissue et al. (2005)	3	Temp Ev Nl and Bl Dc	170.3	−43.2	50	Okarito forest south Westland	NZ	Canopy level	Y	Y
Turnbull et al. (2007)	1	Temp Ev Bl	142.05	−37.03	470	Ballarat	Australia	Defoliation	Y	Y
Warren (2004)	1	Temp Ev Bl	143.53	−37.25	450	Creswick	Australia	N	Y	N
Watanabe et al. (2011)	1	Temp Dc Nl	141	43	180	Asapporo	Japan	CO_2_*N	Y	Y
Wohlfahrt et al. (1999a)	28	Temp C3 grass and forb	11.01	46.01	1540–1900	Monte Bondone	Estern Alps	None	Y	N
Zhang and Dang (2006)	1	Temp Dc Bl	89.14	48.22	200	Ontario	Canada	CO_2_*age	N	Y
Additional datasets										
TRY – Kattge et al. (2011)	1048									
Wullschleger (1993)	110									

* PFT abbreviations: Temp, temperate; Trop, tropical; Ev, evergreen; Dc, deciduous; Nl, needleleaf tree; Bl, broadleaf tree.

*V*_cmax_ and *J*_max_ are calculated by fitting equations 1 and 2, or 1,3, and 4 to sections of the A/C_*i*_ curve (Von Caemmerer and Farquhar 1981; Sharkey et al. 2007), and these calculations are sensitive to the kinetic parameters, *K*_*c*_ and *K*_*o*_ and to Γ_*_, used in the fitting process (Medlyn et al. 2002). Using a method (detailed in Appendix S1) similar to Kattge and Knorr (2007), we removed the variation in *V*_cmax_ and *J*_max_ across studies caused by different parametric assumptions by standardizing *V*_cmax_ and *J*_max_ to a common set of kinetic parameters (derived by Bernacchi et al. 2001). We also corrected *V*_cmax_ and *J*_max_ to a common measurement temperature of 25°C and to the O_2_ partial pressure at the measurement elevation. Errors introduced by the standardization were well within the measurement error of A/C_*i*_ curves (Appendix S2). Standardizing for the kinetic parameters had a substantial impact on *V*_cmax_ and to a lesser extent *J*_max_ (Figure S1), as observed by Kattge et al. (2009). Standardization for O_2_ partial pressure decreases with altitude had a small impact on values taken from plants growing at altitudes up to 2000 m (Figure S2).

We related *J*_max_ and *V*_cmax_ such that:


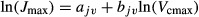
(1)

where *b*_*jv*_ is the slope of the relationship and *a*_*jv*_ the intercept. Gu et al. (2010) demonstrated a method-specific bias on *b*_*jv*_ (on non-log-transformed variables) caused by predetermination of the limitation state of points on the A/C_*i*_. However, most authors in this meta-analysis used a fitting procedure which removed points that were potentially either limitation state (Wullschleger 1993; Sharkey et al. 2007) which minimizes potential biases in *b*_*jv*_.

Where LMA was reported, we converted to SLA by taking the reciprocal of LMA. While this introduced some error (the reciprocal of the mean of a set of values does not equal the mean of the reciprocals of that set), the error was distributed across the whole range of SLA so was unlikely to have biased the effect of SLA. To compare the *J*_max_ to *V*_cmax_ relationship from our dataset, we also used *V*_cmax_ and *J*_max_ data from Wullschleger (1993) and the TRY database (Kattge et al. 2011; data from Atkin et al. 1997; Kattge et al. 2009). *V*_cmax_ and *J*_max_ are measured on a leaf area basis, and in models of photosynthesis, area-based measurement integrates these parameters with light capture. Therefore, we restricted our analysis to leaf-area-based measurements.

### Statistical analysis

To assess the importance of P and SLA as covariates with leaf N in determining *V*_cmax_ and *J*_max_, we developed multiple regressions of *V*_cmax_ or *J*_max_ as the dependent variable and leaf N, leaf P, and SLA as the independent variables. To increase sample size and increase the range of each variable, we also developed multiple regressions of *V*_cmax_ or *J*_max_ against leaf N and either SLA or leaf P. In the analysis of *J*_max_, we also included *V*_cmax_ as an independent variable based on our hypothesis that *W*_*c*_ and *W*_*j*_ are coordinated via the *J*_max_ to *V*_cmax_ relationship. We used linear mixed-model regression framework with leaf traits as fixed effects and the author of the paper from which the data were collected as the random effect (Ordonez et al. 2009). Including the study author as a random effect in the regression model accounted for the nonindependence of data within a study. We were unable to account for differential accuracy between studies, often measured by sampling variance or sample size in meta-analysis, and therefore did not weight the data. All variables were natural-log-transformed to ensure normality of residuals.

Similar to all meta-analyses (Gurevitch and Hedges 1999), there is likely to be some error introduced by the different methods used by the different research groups, but the standardization method and the mixed-model analysis with study group as the random effect will have minimized this error.

All statistical analyses were carried out using the open-source software package R, version 2.13.0 (R Core Development Team 2011). We employed a backward, stepwise, AIC-based model simplification process. Our maximal models contained 3-way interactions for *V*_cmax_ (and all 2-way interactions in the models with two independent variables) and *J*_max_ and were fit with the “lme” function of the “nlme” library (Pinheiro et al. 2011). Models were then simplified using the “dropterm” function of the “MASS” library to conserve marginality (see Venables and Ripley 2002). Model selection aimed to find the minimum adequate model – the model explaining the most variation in the dependent variable with minimum necessary parameters. Model selection was based on the model with the lowest corrected Akaike information criterion (AICc) and with a significance level of each model term of *P* < 0.1, subject to conservation of marginality. The AIC is a relative measure of competing models' likelihood penalized by the number of parameters fit by the model, and the AICc is the AIC when corrected for finite sample size (Burnham and Anderson 2002). Given a set of competing models, the model with the lowest AICc can be considered the preferred model (the minimum adequate model).

We report the likelihood ratio test (LRT) statistic between a model and an intercept only (i.e., only random effects) null model and calculated model significance *P*-values using the chi-square distribution. As there is no mixed-model method to estimate variance in the dependent variable explained by the model, we report the proportional decrease in the residual variance in the minimum adequate model compared with the null, random effects only, model as a metric of explained variance (Xu 2003).

Models were checked for violation of the assumptions of mixed-model linear regression (homoscedasticity of residuals; normal distribution of residuals within the random groups and that observed values of the dependent variable bore a linear relationship to model fitted values), and all minimum adequate models satisfied these checks (a comparison of model assumptions when using nontransformed and transformed data are presented in Appendix S3).

### Modeling carbon assimilation

After Medlyn et al. (2002) and Kattge and Knorr (2007), carbon assimilation was modeled using the Farquhar et al. (1980) biochemical model for perfectly coupled electron transport and the Calvin–Benson cycle, as reported in Medlyn et al. (2002). Enzyme kinetic models of photosynthesis (Farquhar et al. 1980) simulate net CO_2_ assimilation (*A*) as the minimum of the RuBisCO-limited gross carboxylation rate (*W*_*c*_) and the electron transport-limited gross carboxylation rate (*W*_*j*_), scaled to account for photorespiration, minus mitochondrial (dark) respiration (*R*_*d*_). The net assimilation function takes the form:


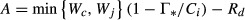
(2)

where Γ_*_ is the CO_2_ compensation point (Pa), the *C*_*i*_ at which the carboxylation rate is balanced by CO_2_ release from oxygenation. Both *W*_*c*_ and *W*_*j*_ are modeled as functions of the intercellular CO_2_ partial pressure (*C*_*i*_ − Pa). *W*_*c*_ follows a Michaelis–Menten function of *C*_*i*_ in which *V*_cmax_ (μmol CO_2_ m^−2^·s^−1^) determines the asymptote:


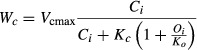
(3)

where *O*_*i*_ is the intercellular O_2_ partial pressure (kPa); *K*_*c*_ and *K*_*o*_ are the Michaelis–Menten constants of RuBisCO for CO_2_ (Pa) and for O_2_ (kPa). The light-limited gross carboxylation rate (*W*_*j*_) is a function of the electron transport rate (*J* - μmol·e·m^−2^·s^−1^) following a similar function of *C*_*i*_ where the asymptote is proportional to *J*:


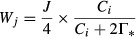


*J* is a function of incident photosynthetically active radiation (*I* – μmol photons m^−2^·s^−1^) that saturates at the maximum rate of electron transport (*J*_max_), formulated by Harley et al. (1992) following Smith (1937), though other formulations exist:


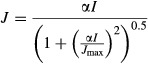


where α is the apparent quantum yield of electron transport (assumed to be 0.24 mol electrons mol^−1^ photons by Harley et al. (1992) although *α* is not invariant in nature) and is the result of multiplying the true quantum yield and light absorption by the leaf. By determining the asymptotes of the two rate-limiting cycles of photosynthesis, it is clear from the above set of equations that carbon assimilation is highly sensitive to *V*_cmax_ and *J*_max_.

Temperature sensitivities of *V*_cmax_ and *J*_max_ were simulated using the modified Arrhenius equation of Johnson et al. (1942), see Medlyn et al. (2002). For consistency with the temperature sensitivity functions of *V*_cmax_ and *J*_max_ (see Medlyn et al. 2002), the temperature sensitivities of the kinetic properties of RuBisCO and the CO_2_ compensation point in the absence of dark respiration were modeled after Bernacchi et al. (2001). See Appendix S4 for further details.

Coefficients of the equations relating *V*_cmax_ to leaf N and *J*_max_ to *V*_cmax_ were taken from the models presented in Table 3. The impact of P and SLA on assimilation was simulated by predicting *V*_cmax_ using the 5th and 95th percentile of either P (0.05 and 0.22 mg·g^−1^) or SLA (adjusted to provide realistic combinations of SLA and leaf N 0.01 m^2^·g^−1^ and 0.025 m^2^·g^−1^) from our database. The biophysical space over which carbon assimilation was simulated was PAR ranging from 0 to 1500 μmol·m^−2^·s^−1^, internal CO_2_ partial pressure of 30 Pa, at two levels of leaf N (0.5 and 3 g·m^−2^) and at a temperature of 25°C.

**Table 3:** Details of the recommended minimum adequate models (MAM) explaining *V*_cmax_ and *J*_max_. All traits were expressed on an area basis and were natural-log-transformed. The LRT was the likelihood ratio test statistic of the model against the null (intercept only) model, and the residual variance reduction was the proportional reduction in residual variance when compared to the null model. A colon represents the interaction between two variables. Using the example of model 1, the equation describing *V*_cmax_ would take the form: ln(*V*_cmax_) = 1.993 + 2.555ln(N) − 0.372ln(SLA) + 0.422ln(N)ln(SLA)

	Response trait	Explanatory traits of the maximal model^1^	Explanatory variables of the MAM	Coefficient	SE	df	Student's *t-test*	*P*	N obs	N groups	Residual variance reduction (%)	LRT	*P*-value
Model 1	*V*_cmax_	N, P	Intercept	3.946	0.229	99	17.26	<0.001	110	8	19.5	25.5	<0.001
			N	0.921	0.301	99	3.06	0.003					
			P	0.121	0.085	99	1.42	0.156					
			N:P	0.282	0.145	99	1.95	0.054					
Model 2	*V*_cmax_	N, SLA	Intercept	1.993	0.410	237	4.86	<0.001	260	20	36.6	99.1	<0.001
			N	2.555	0.522	237	4.89	<0.001					
			SLA	-0.372	0.093	237	-4.00	<0.001					
			N:SLA	0.422	0.115	237	3.67	<0.001					
Model 3	*J*_max_	*V*_cmax_, N, P	Intercept	1.246	0.233	96	5.33	<0.001	105	7	83.5	189.1	<0.001
			*V*_cmax_	0.886	0.043	96	20.60	<0.001					
			P	0.089	0.041	96	2.20	0.033					
Model 4	*J*_max_	*V*_cmax_, N, SLA	Intercept	1.197	0.115	215	10.45	<0.001	235	17	84.2	416.1	<0.001
			*V*_cmax_	0.847	0.025	215	34.23	<0.001					

1 Including all combinations of interactions between each trait.

To simulate the sensitivity of carbon assimilation to the *J*_max_ to *V*_cmax_ slope, the model was driven with a full range of photosynthetically active radiation (PAR, 0–1500 μmol·m^−2^·s^−1^) and three levels of *V*_cmax_ (25, 50 & 90 μmol·m^−2^·s^−1^). For simplicity, we only simulated the sensitivity at 25°C, acknowledging that temperature is also an important factor determining the sensitivity of assimilation to the *J*_max_ to *V*_cmax_ slope.

## Results

### *V*_cmax_ and *J*_max_ in relation to leaf N, leaf P, and SLA

The most likely model, that is, the minimum adequate model, when *V*_cmax_ was regressed on all three leaf traits together (leaf N, P, and SLA) was the model with SLA as the only explanatory variable (see Table S2). However, there were less data available for this analysis (*n* = 90, over 50% of which came from a single study), and as a consequence, the range of leaf N and SLA values were restricted compared with their range in the trivariate models discussed below. For this reason, we present no further discussion of *V*_cmax_ regressed on leaf N, leaf P, and SLA. For *J*_max_ regressed on *V*_cmax_, leaf N, leaf P, and SLA, the minimum adequate model was of *J*_max_ regressed only on *V*_cmax_ and leaf P with no interaction (see Table S2). With increased range in the explanatory variables, we focus on the models with one less explanatory variable.

For *V*_cmax_ regressed against leaf N and either leaf P or SLA, the minimum adequate models were also the maximal models – those with both traits and their interaction (Table 2; models 1 and 2). Models of *V*_cmax_ regressed on leaf N and either SLA or leaf P were both highly significantly different from the null (intercept and random effects only) model (*P* < 0.001).

**Table 2:** Model selection table for multiple regressions of *V*_cmax_ and *J*_max_ regressed against leaf N, or leaf N and *V*_cmax_ respectively, and in combination with either leaf P or SLA. The minimum adequate model (MAM) was the model with the lowest AICc. All traits were expressed on a leaf area basis and were natural-log-transformed

Response trait	Model	Model explanatory variables^1^	Residual variance reduction (%)	AICc
*V*_cmax_	Maximal model, MAM – Model 1	N, P, N:P	19.5	44.2
		N, P	16.6	45.8
		N	13.5	47.7
		P	6.5	56.9
*V*_cmax_	Maximal model, MAM – Model 2	N, SLA, N:SLA	36.6	174.6
		N, SLA	32.5	185.7
		N	30.2	187.8
		SLA	12.3	248.4
*J*_max_	Maximal model	*V*_cmax_, N, P, all 2-way interactions, 3-way interaction	83.6	−115.6
		*V*_cmax_, N, P, all 2-way interactions	83.6	−117.9
		*V*_cmax_, N, P, *V*_cmax_:N, N:P	83.4	−118.9
		*V*_cmax_, N, P, *V*_cmax_:N	83.4	−120.1
		*V*_cmax_, N, P	83.4	−121.4
	MAM – Model 3	*V*_cmax_, P	83.5	−123.2
		*V*_cmax_, P, *V*_cmax_:P	83.5	−121.2
		*V*_cmax_	82.9	−120.8
		N	10.4	49.3
		P	12.5	46.2
*J*_max_	Maximal model	*V*_cmax_, N, SLA, all 2-way interactions, 3-way interaction	85.1	−196.2
		*V*_cmax_, N, SLA, all 2-way interactions	84.7	−193.7
		*V*_cmax_, N, SLA, *V*_cmax_:N, *V*_cmax_:SLA	84.7	−195.3
		*V*_cmax_, N, SLA, *V*_cmax_:SLA	84.6	−194.5
		*V*_cmax_, SLA, *V*_cmax_:SLA	84.5	−196.4
		*V*_cmax_, SLA	84.3	−196.4
	MAM – Model 4	*V*_cmax_	84.2	−196.0
		*V*_cmax_, N, *V*_cmax_:N	84.2	−193.0
		*V*_cmax_, N	84.2	−194.0

1 All models include an intercept term.

**Figure 1:**
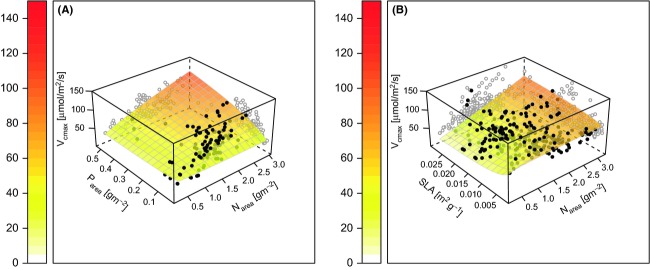
The derived relationships between *V*_cmax_ and leaf nitrogen (Table 3), as modified by leaf P (A – Table 3, model 1) and SLA (B – Table 3, model 2).

For *V*_cmax_ against leaf N and P (model 1), leaf N was a significant explanatory variable (*P* = 0.003), as was the interaction between leaf P and leaf N (*P* = 0.054), although just outside the 95% confidence level (Table 3). The AICc model selection procedure indicates that the P x N interaction was important and the response surface of *V*_cmax_ to leaf N and leaf P (Fig. 1) also shows the importance of leaf P in determining *V*_cmax_. Leaf P modified the relationship of *V*_cmax_ to leaf N such that as leaf P increased, the sensitivity of *V*_cmax_ to leaf N increased (Fig. 1), that is, the coefficient of the interaction term was positive (Table 3). The term for leaf P alone was not significant, but was retained in the minimum adequate model to preserve marginality (see Venables and Ripley 2002).

For *V*_cmax_ against leaf N and P (model 2), increasing SLA increased the sensitivity of *V*_cmax_ to leaf N; however, the magnitude of the effect was smaller than the effect of increasing leaf P (Fig. 1). In contrast to the effect of leaf P, the effect of SLA alone was significant and was contradictory to its effect in interaction – increasing SLA decreased *V*_cmax_ although this effect was only clearly visible at low levels of SLA and leaf N (Fig. 1B). There were few data points at low SLA and low leaf N because as SLA decreases, leaf N concentrations would have to be extremely low to allow low values of leaf N when expressed on an area basis, again suggesting that the effect of SLA on *V*_cmax_ was not substantial.

Leaf P had a larger effect on the *V*_cmax_ to leaf N relationship than did SLA (compare Fig. 1A and B), by contrast SLA was more significant in model 2 than was leaf P in model 1. The contrast arises from the reduced sample size of the leaf P regressions (110 observations in eight groups) compared with the SLA regressions (260 in 20 groups). While the effect of leaf P was greater, statistical confidence in the effect was lower and more data are needed to improve our confidence in the statistical model.

For the multiple regressions of *J*_max_ against *V*_cmax_, N, and P, the minimum adequate model was that of *V*_cmax_ and P, with no interaction term, explaining 84% of the residual variance compared with the null model (Table 2; model 3). For *J*_max_ regressed against *V*_cmax_, N, and SLA, the minimum adequate model was that with *V*_cmax_ alone, explaining 84% of the residual variance when compared to the null model (Table 2; model 4). Both models were highly significantly different from the null model (*P* < 0.001 – Table 3). While model 4 had a slightly higher AICc than the model with *V*_cmax_, SLA and their interaction as model terms (Table 2), SLA and the *V*_cmax_ x SLA interaction were not significant model terms (*P* > 0.1; results not shown). This was also the case for the model with *V*_cmax_ and SLA and this led to the selection of model 4 (*J*_max_ against *V*_cmax_ alone; Tables 2 and 3) as the minimum adequate model. The inclusion of *V*_cmax_ in the regressions of *J*_max_ meant that the traits leaf N, leaf P, and SLA were tested for their effect on *J*_max_ that were orthogonal to their effect already implicitly considered via their effect on *V*_cmax_. The leaf traits were considered as modifiers of the *J*_max_ to *V*_cmax_ relationship, not as direct determinates of *J*_max_.

**Figure 2:**
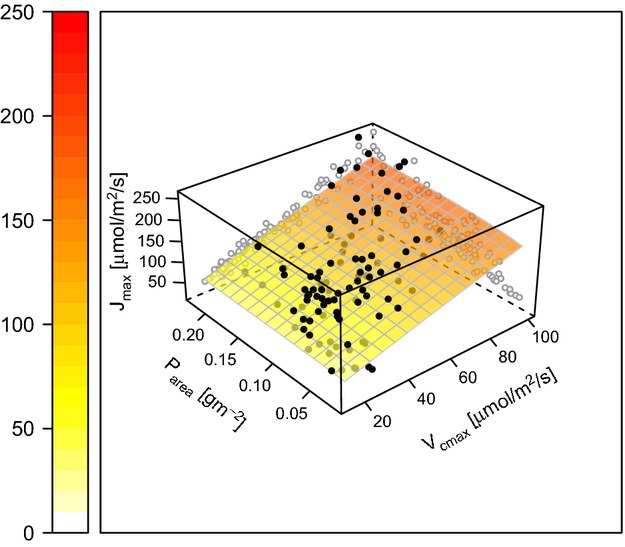
The relationship between *J*_max_ and *V*_cmax_ as modified by leaf P (Table 3, model 3).

**Figure 3:**
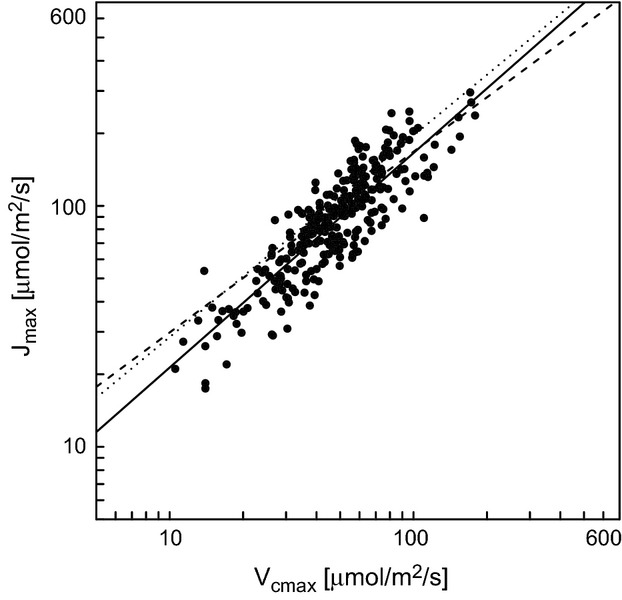
The relationship between *J*_max_ and *V*_cmax_ collected in this study (black circles and solid line) and compared against the regressions based on the Kattge et al. (2009) dataset (dotted line) and the Wullschleger (1993) dataset (dashed line). Log-scaled axes.

The effect of leaf P was significant in model 3; however, variation in leaf P had little effect on calculated values of *J*_max_ (Fig. 2). The effect of *V*_cmax_ was the most important in determining *J*_max_ demonstrating the tight coupling between the two maximum rate parameters. A regression of *J*_max_ on *V*_cmax_ alone yielded 301 observations, with a *b*_*jv*_ of 0.89 ± 0.02 (Table 4). In the first analysis to our knowledge of the in vivo relationship between *J*_max_ and *V*_cmax_, Wullschleger (1993) described a slope coefficient (*b*_*jv*_) of 1.64 for untransformed data. For comparison with our dataset, we natural-log-transformed *J*_max_ and *V*_cmax_ from the Wullschleger (1993) dataset and re-analyzed them with a linear regression. Regression assumptions were not violated by the transformation and *b*_*jv*_ was 0.84 with an *R*^2^ of 0.87 (Table 4). In an analysis of natural-log-transformed *J*_max_ against *V*_cmax_ from the TRY database (Kattge et al. 2011), *J*_max_ scaled against *V*_cmax_ with a *b*_*jv*_ of 0.75 (and *R*^2^ of 0.79). All three datasets have similar slope parameters for the log-transformed relationship ranging from 0.75 for the TRY data to 0.89 for our dataset (Fig. 3).

### Variation in carboxylation rates caused by variation in P and SLA

**Figure 4:**
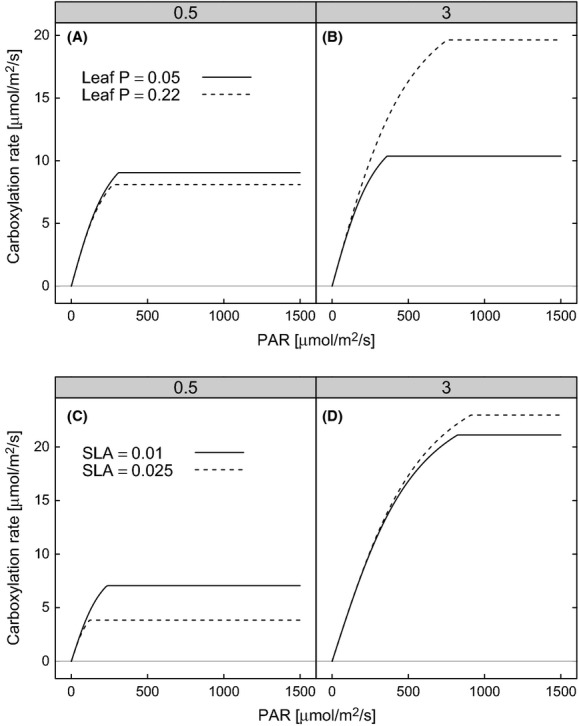
Simulated variation in gross carboxylation light-response curves as a result of variation in leaf P (A–B) or SLA (C–D) used in the minimum adequate models presented in Table 3. Light responses were simulated at two levels of leaf N, 0.5 gm^−2^ (A & C) and 3 gm^−2^ (B & D).

The sensitivity of simulated carboxylation rates to variation in *V*_cmax_ and *J*_max_ caused by variation in leaf P or SLA (based on the minimum adequate models presented in Table 3) is shown in Fig. 4). At high leaf N (3 gm^−2^), increasing leaf P from the 5^th^ to the 95^th^ percentile (0.05 gm^−2^ to 0.22 gm^−2^) almost doubled carboxylation rates at high PAR (Fig. 4), while at low leaf N (0.5 gm^−2^), assimilation was little affected by changes in leaf P. The increase in assimilation caused by increased leaf P at moderate-to-high leaf N, but not at low N, was because leaf P was important only in interaction with N. At low leaf P (0.05 gm^−2^), increasing leaf N from 0.5 to 3 gm^−2^ resulted in a slight increase in carboxylation rates (compare solid lines in Fig. 4A and B). The effect of leaf P on *J*_max_ was so small (Table 3 and Fig. 2) in comparison with the effect of *V*_cmax_ that there was very little effect on carboxylation rates caused by variation in *J*_max_ resulting from variation in leaf P (results not shown).

At high leaf N, increasing SLA from 0.01 m^2^·g^−1^ to 0.025 m^2^·g^−1^ had little effect on simulated carboxylation rates. At low leaf N (0.5 gm^−2^), carboxylation rates were decreased as SLA increased. Assimilation was reduced at low leaf N because the effect of SLA alone (which has a negative relationship to *V*_cmax_) was larger than the effect of SLA in interaction with low levels of leaf N. At higher leaf N, the effect of SLA alone was canceled by the effect of SLA in interaction with leaf N, and therefore, there was little overall effect of SLA on *V*_cmax_ and hence carboxylation rates (Fig. 4).

### The consequence of variation in *b*_*jv*_ on carbon assimilation

**Figure 5:**
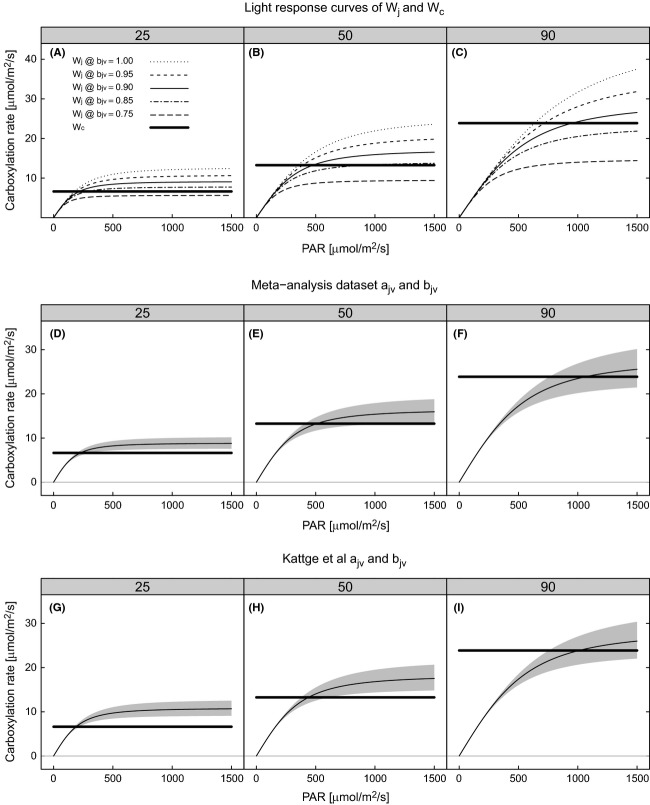
Simulated light-response curves of *W*_*j*_ and *W*_*c*_ in response to b_jv_ variation (A–C), using *a*_*jv*_ and *b*_*jv*_ calculated from the dataset compiled in this study (D–F) and using *a*_*jv*_ and *b*_*jv*_ calculated from the dataset of Kattge et al. (2009) (G–I). All curves calculated at three levels of *V*_cmax_ 25 (A, D & G), 50 (B, E & H), and 90 (C, F, & I) μmol·m^−2^·s^−1^. On panels D–I, the black line within the gray-shaded area represents *W*_*j*_ using the calculated coefficients and the gray-shaded area 95% confidence interval of *W*_*j*_.

To analyze the relationship of *J*_max_ to *V*_cmax_ in more depth, we investigated the effect of the slope parameter (*b*_*jv*_) on the modeled light response of carbon assimilation at three levels of *V*_cmax_ (25, 50, and 90 μmol·m^−2^·s^−1^). Figure 5A–C shows the light-response curves of the *W*_*c*_ and *W*_*j*_ gross carboxylation rates. Obviously, *W*_*c*_ is insensitive to variation in irradiance, and *W*_*j*_ shows the typical saturating response at high light. Increasing *b*_*jv*_ increases the asymptote of *W*_*j*_, which affects the transition point between *W*_*c*_ and *W*_*j*_ limitation. The light level at the transition where *W*_*c*_ and *W*_*j*_ are colimiting increases as *b*_*jv*_ decreases (Fig. 5A–C).

The relationship of the colimiting light level to *b*_*jv*_ allows us to categorize values of *b*_*jv*_ into two types: (1) intermediate values of *b*_*jv*_ where the point of colimitation occurs between the linear phase and the asymptote of the light response; and (2) low values at which there is no colimitation point, that is, electron transport is always limiting. Within the first category, the light level of colimitation is highly sensitive to *b*_*jv*_. At the upper end of these intermediate *b*_*jv*_ values, the colimitation point occurs while assimilation is still in the linear phase of the light response and thus maximizes quantum yield (the differential of the curve), while *W*_*j*_ limits photosynthesis (Fig. 5A–C). At levels of irradiance above the colimitation point, high values of *b*_*jv*_ cause *W*_*j*_ to be substantially higher than *W*_*c*_ representing “spare” electron transport capacity. As *b*_*jv*_ increases, quantum yield decreases and the *W*_*j*_ asymptote approaches the *W*_*c*_ rate of carboxylation. In the second category of *b*_*jv*_ values, the light–response curve asymptotes below the value of *W*_*c*_, that is, assimilation is light limited at all light levels, there is no colimitation, and quantum yield is very low (see Fig. 5c). It is also possible at high values of *b*_*jv*_ for the colimitation point to occur at a fixed level of irradiance, independent of *b*_*jv*_ although these are at values of *b*_*jv*_ > 1 (see Fig. 5A), substantially higher than observed (Table 4).

The *J*_max_ to *V*_cmax_ relationship of the data collected in this study, and those from the TRY database (Table 4), both have values of *b*_*jv*_ within the first category (Fig. 5D–I). The transition is highly dependent on *b*_*jv*_, and the *W*_*c*_ rate of assimilation is generally within the uncertainty of the potential *W*_*j*_ carboxylation rate at saturating light. For the coefficients derived from the data collected in this study, quantum yield is not maximized, that is, the colimitation point is never in the linear phase of the light response. When *V*_cmax_ was 50 μmol·m^−2^·s^−1^ and over, at light levels above those at the colimitation point, *W*_*j*_ was similar but slightly higher than *W*_*c*_. At low photosynthetic capacity (i.e., *V*_cmax_ = 25 μmol·m^−2^·s^−1^) across the whole range of uncertainty, electron transport capacity above that necessary for carboxylation is apparent when *W*_*c*_ is limiting (Fig. 5D and G).

**Table 4:** Slope coefficients from linear regressions of log-transformed *J*_max_ on *V*_cmax_ from the data collected in this study, from the TRY database and from Wullschleger (1993). The data collected in this study were analyzed using a mixed-effects model with the author as the random effect, while data from the other two studies were analyzed using a fixed-effects model

	*N*	Model term	Coefficient	SE	Reduction in residual variance (%)	*P*-value*
This study	301	Intercept	1.010	0.097	86.7	<0.001
		Slope	0.890	0.021		
TRY/Kattge	1048	Intercept	1.668	0.048	78.9	<0.001
		Slope	0.750	0.012		
Wullschleger	110	Intercept	1.425	0.128	87.2	<0.001
		Slope	0.837	0.031		

* For this study's dataset, the *P*-value is based on the LRT statistic, and for Kattge and Wullschleger, it is based on the *F* statistic.

## Discussion

Our goal in this study was to derive relationships of *V*_cmax_ and *J*_max_ in relation to leaf N, P, and SLA. Using a meta-analytic approach to assess patterns among 356 species drawn from 24 different studies around the world, in agreement with many previous studies, we found that *V*_cmax_ increased in relation to leaf N (Wohlfahrt et al. 1999b; Aranda et al. 2006; Bown et al. 2007; Kattge et al. 2009; Domingues et al. 2010) and that both leaf P and SLA increased the sensitivity of *V*_cmax_ to leaf N. We also found that the relationship between *J*_max_ and *V*_cmax_ was not substantially affected by leaf N, leaf P, or SLA (Table 2). Our efforts and in particular the statistical models provide a formal template on which to improve the parameterization of terrestrial ecosystem and biosphere models (TBMs; Tables 3 and 4). We demonstrated the impact of these variable rate parameters in a simple model of photosynthesis.

### Evaluating the three hypotheses

In analyzing the data, we had three a priori hypotheses: (1) leaf P will modify the relationship of *V*_cmax_ to leaf N, (2) leaf P will modify the relationship of *J*_max_ to *V*_cmax_, (3) the relationship between *J*_max_ and *V*_cmax_ results from a trade-off between photosynthetic gain and costs of energy dissipation.

In support of our first hypothesis, we found that leaf P was an important factor modifying the *V*_cmax_ to leaf N relationship. For *V*_cmax_, we recommend the use in TBMs of coefficients and terms of model 1 and model 2 presented in Table 3. For those models, such as CABLE and CLM-CNP, that prognostically simulate, or explicitly parameterize leaf N and leaf P, we recommend the use of model 1 to simulate *V*_cmax_ (Table 3) and we suggest that incorporation of variation in leaf P is necessary for accurate scaling of *V*_cmax_. Many models do not prognostically simulate SLA, and we have demonstrated that while significant, the effect size of SLA on *V*_cmax_ was small and we suggest it is not a priority for inclusion in TBMs for accurate parameterization of *V*_cmax_. However, depending on model structure, SLA is indirectly important for scaling leaf N concentrations to area-based values of leaf N.

In contrast, and with reference to hypothesis two, we find that leaf P had little effect on the *J*_max_ to *V*_cmax_ relationship. For *J*_max_, we recommend the use in TBMs and related tools of the model presented in Table 4 of *J*_max_ regressed on *V*_cmax_ alone. Although the minimum adequate model of *J*_max_ regressed on *V*_cmax_, leaf N and P included leaf P as an explanatory variable, the small coefficients (Table 3) suggested that the additional impact of leaf P on *J*_max_ was minimal as demonstrated in Fig. 3.

The observed relationship between *J*_max_ and *V*_cmax_ run through a chloroplast-level photosynthesis model showed that the *W*_*c*_ rate of assimilation is generally within the uncertainty of the potential *W*_*j*_ carboxylation rate at saturating light and that quantum yield is not maximized. In terms of hypothesis three, the results suggest that the costs of energy dissipation and potential for photoinhibition outweigh the marginal benefits to photosynthetic gain.

### The impact of leaf P

The empirical functions we present can be applied in TBMs with a phosphorus cycle and would allow scaling of *V*_cmax_ and *J*_max_ that will be more in tune with nutrient cycling than using a single parameter value for a particular plant functional type (PFT). The use of the empirical function we developed (model 1, Table 3) will reduce simulated carbon assimilation and productivity by TBMs in regions where leaf P is low and leaf N is high, and should help to improve these simulations (Mercado et al. 2011; Yang et al. 2013). Our finding for leaf P was similar to that of Reich et al. (2009) who found that, in a global analysis, increased leaf P increased the sensitivity of *A*_max_ to leaf N. Reich et al. (2009) showed this modification of the relationship between *A*_max_ and leaf N by leaf P to hold true across biomes with different N/P ratios.

The analysis of *V*_cmax_ and *J*_max_ by Domingues et al. (2010) concluded that leaf N and leaf P were best considered in terms of limiting factors, that is, that *V*_cmax_ was determined by either leaf N or leaf P, as often the interaction term between leaf N and P was not significant. Although within the mixed-model framework we were not able to test the limiting factor hypothesis of Domingues et al. (2010), our results suggest that aggregated across diverse sites and species, there is likely to be some colimitation between N and P.

We also aimed to ascertain whether the effect of leaf P held true across multiple biomes and whether this may be a reason for the different *V*_cmax_ to N sensitivities. There was some suggestion that there was an interaction of biome with the *V*_cmax_ relationship to N and P (results not shown), but the majority of leaf P data were gathered from within the tropical zone (Table 2) and the datasets when divided by biome were dominated by individual studies, reducing the power of the meta-analysis. In data gathered primarily within tropical latitudes, we have shown that leaf P substantially impacts the *V*_cmax_ to leaf N relationship.

Kattge et al. (2009) demonstrated variability in the *V*_cmax_ to leaf N relationship across biomes, indicating that in tropical biomes where P was expected to be more limiting, *V*_cmax_ was less sensitive to leaf N. Our analysis shows that across a range of predominantly tropical biomes, the sensitivity of *V*_cmax_ to N was reduced by low leaf P and the derived relationship may help to move forward from PFT-/biome-based parameterizations in TBMs toward a trait correlation approach.

We demonstrated that variation in *V*_cmax_ related to variation in leaf P had a large impact on carboxylation rates. Increasing leaf P from 0.05 gm^−2^ to 0.22 gm^−2^ approximately doubled modeled gross carboxylation rates under high N levels (Fig. 4). Some of the latest generation of TBMs now includes a P cycle (Wang et al. 2010; Goll et al. 2012; Yang et al. 2013), and Mercado et al. (2011) demonstrated the importance of considering P when simulating carbon fluxes in the Amazon. In addition, anthropogenic N and P pollution has had profound effects on global ecosystems (Penuelas et al. 2012). Evidence suggests that N is more limiting than P in temperate and boreal zones (Elser et al. 2007), which may preclude the measurement of P in these zones or that studies measured P but the effects were not significant so were left out of publications. Despite a comprehensive survey of the literature, assessment of the variation in *V*_cmax_ in relation to the leaf N, leaf P, and SLA remains data limited. To fully quantify the effect of leaf P on the *V*_cmax_ to N relationship, we need more data from all ecosystems, but especially temperate and boreal ecosystems. We appeal to the leaf gas exchange research community to measure leaf P in conjunction with leaf gas exchange across all biomes.

### The impact of SLA

Our results show that the relationship of *V*_cmax_ to leaf N was affected by SLA, albeit a small effect, at low values of leaf N (Fig. 1). Both similar and contrasting effects (Wright et al. 2004; Aranda et al. 2006) in the literature suggest that the effect of SLA on *V*_cmax_ is complex. SLA responds to multiple environmental and ecological factors and leaf density and leaf thickness strongly correlate with leaf N (Niinemets 1999; Poorter et al. 2009). In a previous meta-analysis, the components of SLA – leaf thickness and leaf density – showed different relationships to *A*_max_ (Niinemets 1999), indicating that SLA may not have a consistent effect on photosynthesis. For example, leaf thickness and leaf density are likely to have different effects on internal CO_2_ conductance (*g*_*i*_) and the N allocation ratio between RuBisCO and leaf structural components (Poorter et al. 2009). Unfortunately, with this dataset, we were unable to assess the effect of mesophyll conductance (*g*_*i*_) on the *V*_cmax_ to N relationship. SLA is likely to affect *g*_*i*_ (Flexas et al. 2008), and the effects of SLA on the *V*_cmax_ to N relationship will be best assessed once when variation in *g*_*i*_ can be accounted for.

### Resource allocation between *J*_max_ and *V*_cmax_

The *J*_max_ and *V*_cmax_ relationship represents resource allocation between the two photosynthetic cycles – electron transport and the Calvin–Benson cycle. Coordination of resource investment in photosynthetic capacity is reflected by the strong relationship between *V*_cmax_ and *J*_max_. Given the tight coupling of *J*_max_ with *V*_cmax_ across growth environments and species (Fig. 5), we suggest, as noted in many previous studies (Wullschleger 1993; Beerling and Quick 1995; Harley and Baldocchi 1995; Leuning 1997; Medlyn et al. 2002; Kattge and Knorr 2007), that their coupling may be a fundamental feature of plant photosynthetic trait relationships.

Traditionally, *J*_max_ has been related to *V*_cmax_ based on the assumption that optimization of resource allocation to photosynthesis would maintain a close relationship between these two parameters, an assumption verified by analysis of empirical data (e.g., Wullschleger 1993; Beerling and Quick 1995). The similarity in the regression model parameters between our dataset, the TRY dataset, and that of Wullschleger (1993) was remarkable considering the differences between these datasets (Table 4 & Fig. 3). The Wullschleger (1993) dataset comprised mainly grass and crop species as well as some temperate trees, while our dataset predominantly consists of tropical and temperate tree species.

While the general relationship between *J*_max_ and *V*_cmax_ is preserved across datasets (Fig. 3), there is substantial variation of individual species data from this relationship (Fig. 3). Some of this variation may arise due to the measurement error. *V*_cmax_ and *J*_max_ are differentially sensitive to temperature (Medlyn et al. 2002; Kattge and Knorr 2007), and their temperature sensitivity varies across species (Wohlfahrt et al. 1999b). For most species, this temperature sensitivity is not known, and while necessary, the correction of *V*_cmax_ and *J*_max_ to 25°C with non-species-specific sensitivity parameters may add variation into the *J*_max_ to *V*_cmax_ relationship. *V*_cmax_ is more sensitive to mesophyll conductance than *J*_max_ (Sun et al. 2013) and it may be that some of the variation in the relationship may be attributable to variation in *g*_*i*_; however, it was not possible to determine the effect of *g*_*i*_ with this dataset. We present our results assuming infinite *g*_*i*_ because assuming infinite *g*_*i*_ is currently standard practice in TBMs and was the assumption made by most of the studies used in our meta-analysis. By analyzing the general relationship between *J*_max_ and *V*_cmax_, we aim to provide a framework that can be applied to explain *J*_max_ to *V*_cmax_ relationships and consequences of variation in the relationship.

Maire et al. (2012) demonstrated that plants adjust leaf N investment to coordinate *W*_*c*_ and *W*_*j*_ (Chen et al. 1993) for environmental conditions over the previous month (the lifetime of RuBisCO). Scaling between *J*_max_ and *V*_cmax_, represented by the slope parameter *b*_*jv*_, affects the light (and CO_2_, Von Caemmerer and Farquhar 1981) transition point at which carbon assimilation switches between *W*_*c*_ and *W*_*j*_, that is, the light level where *W*_*c*_ and *W*_*j*_ are colimiting. We hypothesized that *b*_*jv*_ may also coordinate instantaneous *W*_*c*_ and *W*_*j*_ when *W*_*c*_ is limiting as investment in *J*_max_ that would support rates of *W*_*j*_ higher than *W*_*c*_, when *W*_*c*_ is limiting, represents investment in unused resources. At the assumed leaf absorptance and at 25°C, simulations show that potential *W*_*j*_ rates at high light and *W*_*c*_ rates are similar (Fig. 5D–I), when the probable range in *b*_*jv*_ values from our dataset (Table 4) are used. Generally, quantum yield is not maximized. Synthesized across multiple species and environments, the presented relationship suggests that *J*_max_ is related to *V*_cmax_ to coordinate *W*_*j*_ with *W*_*c*_ and hedge against photoinhibition, when RuBisCO carboxylation is limiting. Aggregated across the different species and environments, support for co-ordination at light saturation is a very general assertion. The degree of control that plants have over the relationship between *J*_max_ and *V*_cmax_ needs to be tested in controlled environments at a range of temperature and light levels (Wohlfahrt et al. 1999a) and giving consideration to mesophyll conductance and leaf absorptance.

Maire et al. (2012) show that coordination occurs over monthly timescales, while our simulations (Fig. 5) are on instantaneous timescales. The timescale over which coordination is considered is important, and given the huge diurnal variability in incident light, *W*_*c*_ and *W*_*j*_ cannot always be coordinated on subdaily timescales. The relationship that we derived between *J*_max_ and *V*_cmax_ appears to coordinate, within uncertainty bounds, the *W*_*c*_ and *W*_*j*_ rates of photosynthesis at high light levels (Fig. 5D–I). However, there is some variability and the derived relationship has high W_j_ at low photosynthetic capacity (Fig. 5D and G), and *W*_*j*_ higher than *W*_*c*_ when *W*_*c*_ is limiting indicates unused electron transport capacity at high light. Unused electron transport capacity could produce reducing power not used in carbon reduction and which could be used in biochemical pathways other than the Calvin–Benson cycle (Buckley and Adams 2011) such as the reduction of nitrite to ammonium that occurs in the chloroplast (Anderson and Done 1978; Searles and Bloom 2003) and the production of isoprene (Morfopoulos et al. 2013).

## Conclusion

For the first time, we assess the sensitivity of carbon assimilation to the *J*_max_ to *V*_cmax_ relationship, and results from the meta-analysis suggest that plants may employ a conservative strategy of *J*_max_ to *V*_cmax_ coordination to avoid photoinhibition. Work is needed to extend this analysis with the consideration of mesophyll conductance and species-specific temperature effects.

We also present for the first time the significance of P and SLA on the relationship of *V*_cmax_ to nitrogen and of *J*_max_ to *V*_cmax_ in a globally extensive meta-analysis. Modeling demonstrates that variation in leaf P has large consequences for carbon assimilation. The relationships presented in this study can be used to parameterize *V*_cmax_ and *J*_max_ in a rigorous fashion based on data-derived relationships, moving parameterization away from methods with limited variation or limited grounding in the literature. To fully understand variability in the relationship of *V*_cmax_ and *J*_max_ to leaf N, leaf P, and SLA, work is needed to extend the geographic range of data, particularly into temperate and boreal regions.

## Supporting Information

**Appendix S1** **Table S1.** Species that feature in the meta-analysis **Table S2.** Model selection table for multiple regressions of V_cmax_ and J_max_ regressed against leaf N, P and SLA, or leaf N, P, SLA and V_cmax_ respectively. The minimum adequate model (MAM) was the model with the lowest AICc. All traits were expressed on a leaf area basis and were natural log transformed. **Appendix S2.** Standardisation of V_cmax_ and J_max_ to common kinetic parameters and photosynthetic functions. **Figure S1.** Original and standardised values of *V*_cmax_ and *J*_max_ both expressed at 25^°^C. **Figure S2.** Standardised values of *V*_cmax_ and *J*_max_ (both expressed at 25^°^C) using Eq 9 to calculate *Г*_***_ using *O*_*i*_ assumed by the authors of the original publication (20–21 kPa; *x*-axis) and an assumed reduction in *O*_*i*_ with altitude (*y*-axis). **Appendix S3.** Model assumptions and selection **Table S3.** Model selection table for multiple regressions of *V*_cmax_ and *J*_max_ regressed against leaf N, P and SLA, or leaf N, P, SLA and *V*_cmax_ respectively. **Figure S3.** Plots of the mixed-model regression assumptions for the un-transformed *J*_max_ to *V*_cmax_ relationship for the data collected in this study. **Figure S4.** Plots of the mixed-model regression assumptions for the transformed *J*_max_ to *V*_cmax_ relationship for the data collected in this study. **Appendix 4.** Modelling photosynthesis.
