# Supplementary material for: The relationship of leaf photosynthetic traits – Vcmax and Jmax – to leaf nitrogen, leaf phosphorus, and specific leaf area: a meta-analysis and modeling study
Source: Ecol Evol. 2014 Jul 25;4(16):3218–35. doi: 10.1002/ece3.1173 (PMC4222209; doi:10.1002/ece3.1173)
Supplement: Supplementary file 1 [file ece30004-3218-sd1.docx]

**Appendix S1.**

Table S1. Species that feature in the meta-analysis

| species | author | species | author |
| --- | --- | --- | --- |
|  |  |  |  |
| Acacia dudgeoni | Domingues2010 | Liquidambar styraciflua | Sholtis et al 2004 |
| Acacia gourmaensis | Domingues2010 | Maytenus senegalensis | Domingues2010 |
| Acacia senegal | Domingues2010 | Meterosideros umbellata | Tissue et al 2005 |
| Acer rubrum | Bauer et al 2001 | Metrosideros umbellata | Carswell et al 2005 |
| Acer saccharum | Kubiske et al 2002 | Mikania micrantha | Deng et al 2004 |
| Achillea millefolium | Wohlfahrt 1999 | Mikania cordata | Deng et al 2004 |
| Adansonia digitata | Domingues2010 | Nardus stricta | Wohlfahrt 1999 |
| Agrostis tenuis | Wohlfahrt 1999 | Nesogordonia papaveriferum | Domingues2010 |
| Albizia ferruginea | Domingues2010 | Parinari congoensis | Domingues2010 |
| Alchemilla vulgaris | Wohlfahrt 1999 | Parinari polyandra | Domingues2010 |
| Anogeissus leiocarpus | Domingues2010 | Phaseolus vulgaris | Bruck et al 2006 |
| Antiaris africana | Domingues2010 | Picea abies | Merilo et al 2006 |
| Arnica montana | Wohlfahrt 1999 | Picea rubens | Bauer et al 2001 |
| Avenella flexuosa | Wohlfahrt 1999 | Pinus densiflora | Han et al 2008 |
| Betula alleghaniensis | Bauer et al 2001 | Pinus pinaster | Porte and Loustau 1998 |
| Betula papyrifera | Zhang and dang 2006 | Pinus radiata | Bown 2007 |
| Blighia sapida | Domingues2010 | Pinus strobus | Bauer et al 2001 |
| Bosqueia angloensis | Domingues2010 | Pinus sylvestris | Jach and Ceulemans 2000 |
| Briza media | Wohlfahrt 1999 | Plantago atrata | Wohlfahrt 1999 |
| Calluna vulgaris | Wohlfahrt 1999 | Plantago media | Wohlfahrt 1999 |
| Campanula scheuchzeri | Wohlfahrt 1999 | Polygonum viviparum | Wohlfahrt 1999 |
| Chromolaena odorata | Domingues2010 | Populus tremuloides | Kubiske et al 2002 |
| Cochlospermum planchoni | Domingues2010 | Populus x euramericana | Calfapietra et al 2005 |
| Cola caricifolia | Domingues2010 | Potentilla aurea | Wohlfahrt 1999 |
| Cola gigantea | Domingues2010 | Prumnopitys ferruginea | Carswell et al 2005 |
| Combretum ghasalense | Dominguez2010 | Pseudotsuga menziezii | Manter 2005 |
| Combretum glutinosum | Domingues2010 | Pterocarpus erinaceus | Domingues2010 |
| Combretum micranthum | Domingues2010 | Pulsatilla sulphurea | Wohlfahrt 1999 |
| Corymbia aparrerinja | Cernusak2011 | Pyncanthus angloensis | Domingues2010 |
| Corymbia latifolia | Cernusak2011 | Quercus petraea | Rodriguez Calcerrada et al 2008 |
| Corymbia terminalis | Cernusak2011 | Quercus pyrenaica | Rodriguez Calcerrada et al 2008 |
| Corynanthe pachycaras | Domingues2010 | Quercus rubra | Bauer et al 2001 |
| Dacrydium cupressinum | Carswell et al 2005 | Quercus suber | Aranda et al 2005 |
| Dacrydium cupressinum | Tissue et al 2005 | Quintinia acutifolia | Tissue et al 2005 |
| Dactylis glomerata | Wohlfahrt 1999 | Ranunculus acris | Wohlfahrt 1999 |
| Daniellia oliveri | Domingues2010 | Rhinanthus alectorolophus | Wohlfahrt 1999 |
| Daphniphyllum humile | Katahata 2007 | Rumex alpestris | Wohlfahrt 1999 |
| Detarium senegalense | Domingues2010 | Sesleria varia | Wohlfahrt 1999 |
| Eucalyptus coolabah | Cernusak2011 | Species | Author |
| Eucalyptus globulus | Turnbull et al 2007 | Terminalia avicennioides | Domingues2010 |
| Eucalyptus globulus Labill. | Warren 2004 | Terminalia glaucescens | Domingues2010 |
| Eucalyptus grandis | Grassi et al 2002 | Terminalia glaucescens | Domingues2010 |
| Eucalyptus miniata | Cernusak2011 | Terminalia laxiflora | Domingues2010 |
| Eucalyptus pruinosa | Cernusak2011 | Terminalia macroptera | Domingues2010 |
| Eucalyptus tectifica | Cernusak2011 | Trifolium alpinum | Wohlfahrt 1999 |
| Eucalyptus tetrodonta | Cernusak2011 | Trifolium montanum | Wohlfahrt 1999 |
| Festuca rubra | Wohlfahrt 1999 | Trifolium pratense | Wohlfahrt 1999 |
| Ficus saussureana | Domingues2010 | Trilepsium madagascariense | Domingues2010 |
| Geranium sylvaticum | Wohlfahrt 1999 | Triplochiton scleroxylon | Domingues2010 |
| Holarrhena floribunda | Domingues2010 | Trollius europaeus | Wohlfahrt 1999 |
| Hymenostegia afzelii | Domingues2010 | Tsuga canadensi | Bauer et al 2001 |
| Khaya anthotheca | Domingues2010 | Vaccinium myrtillus | Wohlfahrt 1999 |
| Koeleria pyramidata | Wohlfahrt 1999 | Vaccinium uliginosum | Wohlfahrt 1999 |
| Lannea acida | Domingues2010 | Vitellaria paradoxa | Domingues2010 |
| Lannea kersiingii | Domingues2010 | Vitex granifolia | Domingues2010 |
| Larix gmelinii x kaempferi | Watanabe2011 | Weinmannia racemosa | Carswell et al 2005 |
| Leucadendron xanthoconus | Midgley et al 1999 | Weinmannia racemosa | Tissue et al 2005 |
|  |  |  |  |

Table A2. Sources of data collected for the meta-analysis and associated information including location, number of species and any experimental treatment.

| Reference | | Number of species | | PFT* | | | Longitude (^o^E) | | Latitude (^o^N) | | Elevation (m) | location | | | country | | experiment | | N | P |
| --- | --- | --- | --- | --- | --- | --- | --- | --- | --- | --- | --- | --- | --- | --- | --- | --- | --- | --- | --- | --- |
| Aranda et al. 2005 | 1 | | Temp Ev Bl | | -3.43 | 39.23 | | 650 | | alburquerque | | | Spain | light*water | | y | | n | | |
| Bauer et al. 2001 | 6 | | Temp Dc Bl & Ev Nl | | -71.03 | 42.21 | | 40 | | havard forest | | | USA | CO_2_*N | | y | | n | | |
| Bown et al. 2007 | 1 | | Temp Ev Nl | | 176.13 | -38.26 | | 600 | | Purokohukohu Experimental Basin | | | NZ | N*P | | y | | y | | |
| Br$ü$ck & Guo 2006 | 1 | | Temp legume crop | | 10.08 | 54.19 | | 40 | | Kiel | | | Germany | NH_4_ vs NO_3_ | | y | | n | | |
| Calfapietra 2005 | 1 | | Temp Dc Bl | | 11.48 | 42.22 | | 150 | | Viterbo | | | Italy | CO_2_*N canopy level | | y | | n | | |
| Carswell et al. 2005 | 4 | | Temp Dc Bl & Ev Nl | | 170.3 | -43.2 | | 90 | | Okarito | | | NZ | N*P | | y | | y | | |
| Cernusak et al. 2011 | 2 | | Trop Ev Bl | | 139.56 | -22.59 | | 150 | | Boulia | | | Australia | none | | y | | y | | |
| Cernusak et al. 2011 | 2 | | “ | | 133.19 | -17.07 | | 230 | | Sturt plains | | | Australia | none | | y | | y | | |
| Cernusak et al. 2011 | 2 | | “ | | 132.22 | -15.15 | | 170 | | Dry creek | | | Australia | none | | y | | y | | |
| Cernusak et al. 2011 | 2 | | “ | | 131.23 | -14.09 | | 70 | | Daly river | | | Australia | none | | y | | y | | |
| Cernusak et al. 2011 | 2 | | “ | | 131.07 | -13.04 | | 80 | | Adelaide river | | | Australia | none | | y | | y | | |
| Cernusak et al. 2011 | 2 | | “ | | 131.08 | -12.29 | | 40 | | Howard springs | | | Australia | none | | y | | y | | |
| Deng 2004 | 2 | | Sub-trop forb | | 113.17 | 23.08 | | 10 | | Guanzhou | | | China | none | | y | | n | | |
| Domingues et al 2010 | 3 | | Trop Dc Bl | | -1.5 | 15.34 | | 280-300 | | Hombori | | | Mali | none | | y | | y | | |
| Domingues et al 2010 | 7 | | “ | | -1.17 | 12.73 | | 250 | | Bissiga | | | Burkina Faso | none | | y | | y | | |
| Domingues et al 2010 | 8 | | “ | | -3.15 | 10.94 | | 300 | | Dano | | | Burkina Faso | none | | y | | y | | |
| Domingues et al 2010 | 5 | | “ | | -1.86 | 9.3 | | 370 | | Mole | | | Ghana | none | | y | | y | | |
| Domingues et al 2010 | 8 | | “ | | -1.18 | 7.3 | | 170 | | Kogye | | | Ghana | none | | y | | y | | |
| Domingues et al 2010 | 21 | | Trop Dc Bl & Ev Bl | | -1.7 | 7.72 | | 200 | | Boabeng Fiame | | | Ghana | none | | y | | y | | |
| Domingues et al 2010 | 4 | | “ | | -2.45 | 7.14 | | 25 | | Asukese | | | Ghana | none | | y | | y | | |
| Grassi 2002 | 1 | | Sub-trop Ev Bl | | 149.07 | -35.18 | | 600 | | Canberra | | | Australia | N | | y | | n | | |
| Han et al. 2008 | 1 | | Temp Ev Nl | | 138.8 | 35.45 | | 1030 | | Canberra | | | Australia | N | | y | | n | | |
| Katahata et al 2007 | 1 | | Ev shrub | | 138.4 | 36.51 | | 900 | | Niigata | | | Japan | light*leaf age | | y | | n | | |
| Kubiske 2002 | 2 | | Temp Bl Dc | | -84.04 | 45.33 | | 215 | | Pellston | | | USA | N* CO_2_*light | | y | | n | | |
| Manter 2005 | 1 | | Temp Ev Nl | | -122.4 | 45.31 | | 75 | | Portland | | | USA | N | | y | | n | | |
| Merilo et al 2006 | 2 | | Temp Ev Nl | | 26.55 | 58.42 | | 65 | | Saare | | | Estonia | light | | y | | n | | |
| Midgley et al 1999 | 4 | | Temp Ev shrub | | 20 | -34.5 | | 120 | | Cape Agulhas | | | SA | CO_2_*N&P | | y | | n | | |
| Porte & Lousteau 1998 | 1 | | Temp Ev Nl | | -0.46 | 44.42 | | 60 | | Bordeaux | | | France | leaf age*canopy level | | y | | y | | |
| Rodriguez-Calcerrada et al. 2008 | 2 | | Temp Dc Bl | | -3.3 | 41.07 | | 50 | | Madrid | | | Spain | light | | y | | n | | |
| Sholtis 2004 | 1 | | Temp Dc Bl | | -84.2 | 35.54 | | 230 | | Oak Ridge | | | USA | CO_2_*canopy level | | y | | n | | |
| Tissue et al. 2005 | 3 | | Temp Ev Nl & Bl Dc | | 170.3 | -43.2 | | 50 | | Okarito forest south Westland | | | NZ | canopy level | | y | | y | | |
| Turnbull et al. 2007 | 1 | | Temp Ev Bl | | 142.05 | -37.03 | | 470 | | Ballarat | | | Australia | defoliation | | y | | y | | |
| Warren 2004 | 1 | | Temp Ev Bl | | 143.53 | -37.25 | | 450 | | Creswick | | | Australia | N | | y | | n | | |
| Watanabe et al. 2011 | 1 | | Temp Dc Nl | | 141 | 43 | | 180 | | Asapporo | | | Japan | CO_2_*N | | y | | y | | |
| Wohlfahrt et al. 1999a | 28 | | Temp C3 grass & forb | | 11.01 | 46.01 | | 1540-1900 | | Monte Bondone | | | Estern Alps | none | | y | | n | | |
| Zhang & Dang 2006 | 1 | | Temp Dc Bl | | 89.14 | 48.22 | | 200 | | Ontario | | | Canada | CO_2_*age | | n | | y | | |
|  |  | |  | |  |  | |  | |  | | |  |  | |  | |  | | |
| **Additional Datasets** |  | |  | |  |  | |  | |  | | |  |  | |  | |  | | |
| TRY – Kattge et al 2011 | 1048 | |  | |  |  | |  | |  | | |  |  | |  | |  | | |
| Wullschleger 1993 | 110 | |  | |  |  | |  | |  | | |  |  | |  | |  | | |
|  |  | |  | |  |  | |  | |  | | |  |  | |  | |  | | |

* PFT abbreviations: Temp – temperate, Trop – tropical, Ev – evergreen, Dc – deciduous, Nl – needleleaf tree, Bl – broadleaf tree

**Appendix 2.** Standardisation of V_cmax_ and J_max_ to common kinetic parameters and photosynthetic functions.

For carboxylation limited photosynthesis, Eq 1 shows assimilation (*A*) plus dark respiration (*R_d_*) as a function of internal CO_2_ partial pressure (*C_i_*; Pa), the Michaelis-Menten kinetic parameters (*K_c_*; Pa and *K_o_*; kPa), the CO_2_ compensation point in the absence of *R_d_* (*Г_*_*; Pa), the inter-cellular O_2_ partial pressure (*O_i_*; kPa) and the maximum rate of carboxylation (*V_cmax_*; µmol C m^-2^ s^-1^):

$A+R_{d}=V_{c\max}\frac{C_{i}-\Gamma_{*}}{C_{i}+K_{c}\left(1+\frac{O_{i}}{K_{o}}\right)}$ (A1)

Eq 1 is fitted to *A*/*C_i_* data and used to calculate *V_cmax_*. The calculated value of *V_cmax_* is dependent on the value of the parameters *K_c_* and *K_o_* and *Г_*_* and different studies use different values of these parameters. We standardised the values of *V_cmax_* and *J_max_* using the method described below and assuming values of *K_c_* and *K_o_* and *Г_*_* after Bernacchi *et al.*, (2001). The reference from which the original authors took their parameters are given in Table S1. The standardisation method is based on the principle that assimilation (*A*) plus dark respiration (*R_d_*) must be equal for both the original parameter set and the standardised parameter set, such that:

$V_{cmax}^{o}g^{o}\text{ = V}_{cmax}^{s}g^{s}$, (A2)

where the superscript *o* refers to the original parameters and *s* to the standardised parameters, and:

$g\text{ = }\frac{C_{i}-Г_{*}}{C_{i}+K_{c}\left(\text{1+ }\frac{O_{i}}{K_{o}}\right)}$ , (A3)

Eq 2 cannot be simply rearranged to find *V^s^_cmax_* as that would leave *V^s^_cmax_* a function of *C_i_* and *V_cmax_* must be invariant with *C_i_*. The solution is to find a value of *V^s^_cmax_* that minimises the difference between *A* + *R_d_* calculated using the original parameter sets and *A* + *R_d_* calculated using the standard parameter sets, at a number of *C_i_* values (at which *V_cmax_* would be expected to be limiting). In other words, to find a value of *V^s^_cmax_* that minimises the following function:

$f\left(V_{cmax}^{s}\right)\text{ = }\sum_{j=1}^{n} \left(V_{cmax}^{o}g_{j}^{o}\text{ - }V_{cmax}^{s}g_{j}^{s}\right)^{2}$ , (A4)

where *j* is the *j*th *C_i_* value used in calculating *g*. The differential of Eq 4 is:

$f' = \sum_{j=1}^{n} 2g_{j}^{s}\left(V_{cmax}^{o}g_{j}^{o}\text{ - }V_{cmax}^{s}g_{j}^{s}\right)$ , (A5)

set Eq 4 to equal 0 and solved for *V^s^_cmax_*, is:

$V_{cmax}^{s}\text{ = }\frac{V_{cmax}^{o}\sum_{j=1}^{n} g_{j}^{o}g_{j}^{s}}{\sum_{j=1}^{n} \left(g_{j}^{s}\right)^{2}}$, (A6)

This method can also be applied to standardising *J_max_* but involves additional steps to find the original rate of electron transport (*J* - µmol e m^-2^ s^-1^); then find the standardised *J* (*J^s^*) based on Eq 6 but substituting *V^o^_cmax_* and *V^s^_cmax_* for *J^o^* and *J^s^* ; and substituting *g* for *h*, where *h* is (assuming the Harley *et al*., 1992, and many previously, function):

$h = \frac{C_{i}-\Gamma_{*}}{4\left(C_{i}+2\Gamma_{*}\right)}$ (A7)

*J^s^_max_* can then be calculated by rearranging the function that calculates *J* as a function of *J_max_* and substituting in *J^s^*. *J* was calculated from *J_max_* using the original function of *J_max_* reported in the paper and then standardised *J_max_* was calculated using the (Harley *et al.*, 1992) formulation after (Smith, 1937) rearranged for *J_max_*, as this was the function used by many of the authors of the original studies:

$J_{max} = \frac{\alpha^{2}I^{2}}{\left[\left(\frac{\alpha I}{J}\right)^{2}-1\right]^{0.5}}$, (A8)

where *α* is the apparent quantum yield on an incident light basis (mol electrons per mol photons), assumed to be 0.24 (Harley *et al.*, 1992); and leaf *I* is photosynthetic photon flux density (μmol m^-2^s^-1^).

The standardisation process involves additional steps so that initially the original values of *V_cmax_* and *J_max_* were converted back to their measurement temperature (using the method described in the original study). Then the values of *V_cmax_* and *J_max_* were adjusted to values at 25^o^C using the modified Arrhenius equation of Johnson *et al.*, (1942) and see Medlyn *et al.*, (2002), that accounts for a drop in enzymatic efficiency above a temperature optimum. Parameters for the modified Arrhenius equation were taken from Bunce, (2000) and Medlyn *et al.*, (2002). Where temperature sensitivities were measured we used these and the functions from the original papers. The standardisation was then conducted for all values at 25^o^C (see Figure S1 for a comparison of original and standardised parameters both expressed at 25^o^C).

All studies assumed O_2_ partial pressure (*O_i_*) to be 20-21 kPa. However, some studies measured *A*/*C_i_* curves at altitudes where *O_i_* were likely to be substantially lower than assumed. We accounted for the differences in the assumed *O_i_* and the actual *O_i_* caused by altitude on the estimation of *V_cmax_* and *J_max_*, by assuming a 10% decrease in *O_i_* every 1000m altitude. It was necessary to account for *O_i_* in Eq 1 and 3 it was also necessary to account for the effect of *O_i_* on photorespiration and hence the CO_2_ compensation point in the absence of dark respiration (*Г_*_*). Commonly *Г_*_* is determined as a function of temperature (Bernacchi *et al.*, 2001; Medlyn *et al.*, 2002; Sharkey *et al.*, 2007). However, the temperature functions of *Г_*_* are unable to account for changes in *O_i_* and so for the *O_i_* we revert to the (Caemmerer & Farquhar, 1981) equation to calculate *Г_*_* as a function of :

$Г_{*}=\frac{\text{0.5V}_{omax}K_{c}O_{i}}{V_{cmax}K_{o}}$ (A9)

where *V_omax_* is the maximum rate of oxygenation by RuBisCO. We assumed a *V_omax_* to *V_cmax_* ratio of 0.21 (Farquhar *et al.*, 1980).

The use of Eq 9 to calculate *Г_*_* also introduced a change in *V_cmax_* values that was not simply a result of correcting for *O_i_* but also a result of the function itself. To account for this we also standardised *V_cmax_* using Eq 9 to calculate *Г_*_* but assuming an *O_i_* of 20 kPa. The results of these two standardisations are shown in Figure S2 and standardising for *O_i_* had a relatively small effect on *V_cmax_* and *J_max_*. The minor impact of altitudinal variation of *O_i_* on calculation of *V_cmax_* was also found by (Wohlfahrt *et al.*, 1999; Kattge & Knorr, 2007), so for our analysis we standardised *V_cmax_* and *J_max_* by calculated *Г_*_* as a function of temperature and assuming an *O_i_* of 20 kPa.

The error introduced by the standardisation could be estimated by calculating the root mean square error in *A* plus *R_d_* between the original parameter set and the standardised parameter set at several vales of *C_i_* (Eq 4). The inter-quartile range of the root mean square error in *A* plus *R_d_* was 0.06-0.35 μmol m^-2^s^-1^ for *V_cmax_* and 0.03-0.13 for *J_max_*, well within the range of assimilation measurement error.


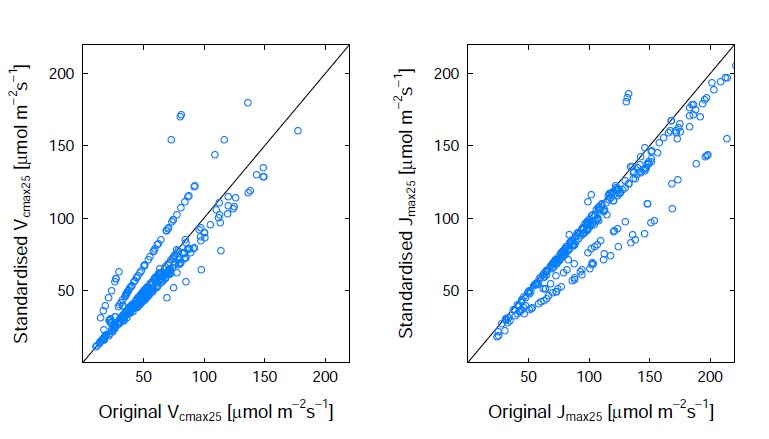


Figure A1. Original and standardised values of *V_cmax_* and *J_max_* both expressed at 25^o^C.


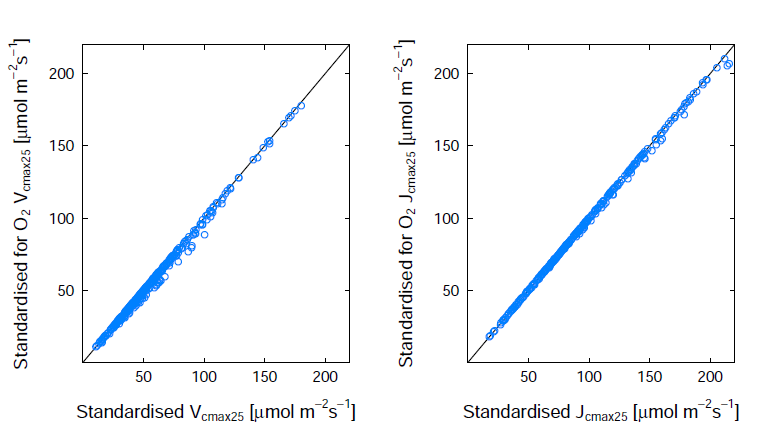


Figure A2. Standardised values of *V_cmax_* and *J_max_* (both expressed at 25^o^C) using Eq 9 to calculate *Г_*_* using *O_i_* assumed by the authors of the original publication (20-21 kPa; x-axis) and an assumed reduction in *O_i_* with altitude (y-axis).

**Appendix 3.** Model assumptions and selection

Table A3. Model selection table for multiple regressions of V_cmax_ and J_max_ regressed against leaf N, P and SLA, or leaf N, P, SLA and V_cmax_ respectively. The minimum adequate model (MAM) was the model with the lowest AICc. All traits were expressed on a leaf area basis and were natural log transformed.

| Response trait | Model | Model explanatory variables* | AICc |
| --- | --- | --- | --- |
|  |  |  |  |
| V_cmax_ | Maximal model | N,P,SLA, all 2-way ints, 3 way int | 44.72 |
|  |  | N,P,SLA, all 2-way ints | 42.21 |
|  |  | N,P,SLA, N:P, P:SLA | 40.39 |
|  |  | N,P,SLA, N:P | 40.45 |
|  |  | N,P,SLA | 41.46 |
|  |  | N,SLA,N:SLA | 40.67 |
|  |  | N | 47.31 |
|  |  | P | 52.29 |
|  | MAM | SLA | 39.43 |
|  |  |  |  |
| J_max_ | Maximal model | V_cmax_,N,P,SLA, all 2-way ints, all 3-way ints, 4-way int | -89.43 |
|  |  | V_cmax_,N,P,SLA, all 2-way ints (-V_cmax_:N), V_cmax_:P:SLA, N:P:SLA | -98.69 |
|  |  | V_cmax_,N,P,SLA, all 2-way ints | -86.29 |
|  |  | V_cmax_,N,P,SLA | -98.48 |
|  | MAM | V_cmax_,P | -99.51 |
|  |  | V_cmax_,N,SLA | -98.64 |
|  |  | V_cmax_ | -98.54 |
|  |  |  |  |

* all models include an intercept term.

Figure A3. Plots of the mixed-model regression assumptions for the un-transformed J_max_ to V_cmax_ relationship for the data collected in this study. Top left – model residuals against model fitted values, there should be no pattern to the residuals, note the increasing variance in the residuals with as the fitted values increase (heteroscedasticity). Bottom left – J_max_ against model fitted values of J_max_, relationship should be linear, compare with Figure S3 below. Panels on the right – the distribution of residuals within a subset of the author groups – should be normally distributed, compare with Figure S3 below.

Figure A4. Plots of the mixed-model regression assumptions for the transformed J_max_ to V_cmax_ relationship for the data collected in this study. Top left – model residuals against model fitted values, note the even distribution of the residuals as the fitted values increase. Bottom left – J_max_ against model fitted values of J_max_, relationship should be linear. Panels on the right – the distribution of residuals within a subset of the author groups – should be normally distributed.

**Appendix 4.** Modelling photosynthesis

After Medlyn et al. (2002) and Kattge and Knorr (2007), carbon assimilation was modelled using the (Farquhar et al. 1980) biochemical model for perfectly coupled electron transport and Calvin-Benson cycle, as refined by Farquhar and von Caemmerer (1982). Net CO_2_ uptake (*A*) is modelled as the minimum of the RuBisCO limited assimilation rate (*W_c_*) and the electron transport limited assimilation rate (*W_j_*) minus mitochondrial respiration (*R_d_*). Both *W_c_* and *W_j_* are modelled as functions of the inter-cellular CO_2_ concentration (*C_i_* - µmol mol^-1^) minus the CO_2_ compensation point (µmol mol^-1^) where the RuBisCO carboxylation velocity and half the RuBisCO oxygenation velocity are equal, i.e. net carbon assimilation is zero.

$A=min\left\{W_{c},W_{j}\right\}\left(1-\Gamma_{\text{*}}/C_{i}\right)-R_{d}$ (A1)

*W_c_* follows a Michaelis-Menton function of *C_i_* where *V_cmax_* (µmol C m^-2^ s^-1^) determines the asymptote:

$W_{c}=V_{cmax}\frac{C_{i}}{C_{i}+K_{c}\left(1+\frac{O_{i}}{K_{o}}\right)}$ (A2)

where *O_i_* is the inter-cellular O_2_ concentration (mmol mol^-1^); *K_c_* is the Michaelis-Menton constant for CO_2_ (µmol mol^-1^) and *K_o_* is the Michaelis-Menton constant for O_2_ (mmol mol^-1^). The rate of electron transport (*J* - µmol e m^-2^ s^-1^) is converted to the light limited carboxylation rate (*W_j_*) following a similar Michaelis-Menton function of *C_i_* where the asymptote is a function of *J*:

$W_{j}=\frac{J}{4}\times\frac{C_{i}}{C_{i}+2\Gamma_{\text{*}}}$ (A3)

*J* is a function of incident photosynthetically active radiation (*I* – µmol photons m^-2^ s^-1^) that saturates at the maximum rate of electron transport (*J_max_*):

$J=\frac{\alpha I+J_{max}-\sqrt{\left(\alpha I+J_{max}\right)^{2}-\text{4θα}IJ_{max}}}{2\theta}$ (A4)

where *α* is the quantum yield of electron transport (0.3 mol electrons mol^-1^ photons), and *θ* is the curvature of the light response curve.

Temperature sensitivities of V_cmax_ and J_max_ were simulated using the modified Arrhenius equation of (Johnson, Eyring & Williams 1942, see Medlyn et al. 2002) further modified by Kattge and Knorr (2007) to make the temperature optima a function of growth temperature.

$k=k_{25}\text{exp}\left[\frac{H_{a}\left(T_{k}-298\right)}{298RT_{k}}\right]\frac{1+\text{exp}\left[\frac{298\Delta S-H_{d}}{298R}\right]}{1+\text{exp}\left[\frac{T_{k}\Delta S-H_{d}}{T_{k}R}\right]}$ (A5)

where k_25_ is the value of V_cmax_ or J_max_ at 25^o^C; T_k_ is the environmental temperature (K); H_a_ and H_d_ are the activation and deactivation energy and R is the universal gas constant. *f*(t_growth_) gives the value of ΔS (Medlyn et al. 2002) as a function of growth temperature after Kattge and Knorr (2007):

$\Delta S=a_{\Delta S,x}+b_{\Delta S,x}t_{growth}$ (A6)

where a_ΔS,x_ and b_ΔS,x_ are constants and t_growth_ (^o^C) is the growth temperature. For consistency with the temperature sensitivity functions of V_cmax_ and J_max_ (see Medlyn et al. 2002), the temperature sensitivities of the kinetic properties of RuBisCO and the CO_2_ compensation point in the absence of dark respiration were modelled after Benacchi et al. (2001):

$K_{c}=\text{404.9exp}\left[\frac{79430\left(T_{k}-298\right)}{\text{298R}T_{k}}\right]$ (A7)

$K_{o}=\text{278.4exp}\left[\frac{36380\left(T_{k}-298\right)}{\text{298R}T_{k}}\right]$ (A8)

and:

$\Gamma_{\text{*}}=\text{42.75exp}\left[\frac{37830\left(T_{k}-298\right)}{\text{298R}T_{k}}\right]$ (A9)
